# Rectus Sheath Hematoma in COVID-19 Patients as a Mortal Complication: A Retrospective Report

**DOI:** 10.1155/2022/7436827

**Published:** 2022-02-12

**Authors:** Hossein Zabihi Mahmoudabadi, Azar Hadadi, Mohammad Reza Fattahi, Samira Kafan, Mohammad Ashouri, Rashad Allahbeigi, Reza Hajebi

**Affiliations:** ^1^Department of Surgery, Sina Hospital, Tehran University of Medical Sciences, Tehran, Iran; ^2^Department of Infectious Diseases, Sina Hospital, Tehran University of Medical Sciences, Tehran, Iran; ^3^Virology Research Center, Tehran University of Medical Sciences, Tehran, Iran; ^4^Department of Pulmonary Disease, Sina Hospital, Tehran University of Medical Sciences, Tehran, Iran; ^5^Department of Surgery, Imam Khomeini Hospital, Tehran University of Medical Sciences, Tehran, Iran

## Abstract

**Background:**

Rectus sheath hematoma is a rare self-limited presentation that has become a concern in hospitalized COVID-19 patients receiving anticoagulant therapies.

**Method:**

A retrospective multicentric study was conducted in referral hospitals affiliated with the Tehran University of Medical Science, Tehran, Iran, between June and August 2021. Patients with a confirmed diagnosis of COVID-19 that were complicated with rectus sheath hematoma during hospitalization were included. Median (lower quartile to upper quartile) was used to report the distribution of the results.

**Result:**

This study was conducted on nine patients with confirmed COVID-19 pneumonia, including eight females and one male. The severity of viral pneumonia was above average in eight patients. The median age and median body mass index were 65 (55.5 to 78) years and 29.38 (23.97 to 31.71) kg/m^2^. The duration of anticoagulant therapy was 10 (6 to 14) days, and the median length of hospital stay was 20 (10 to 23.5) days. Rectus sheath hematoma occurred after a median reduction of 4 (2.7 to 6.6) units in blood hemoglobin. Although 66.7% received ICU care and all of them were under full observation in well-equipped hospitals, the mortality rate was 55.6%.

**Conclusion:**

In summary, increased levels of inflammatory markers such as lactic acid dehydrogenase along with an abrupt decrease in blood hemoglobin in COVID-19 patients should be considered as predisposing factors for rectus sheath hematoma, especially in patients with moderate to severe COVID-19 pneumonia under anticoagulant therapy. This complication had been considered a self-limited condition; however, it seems to be fatal in patients with COVID-19 pneumonia. Further studies in larger sample sizes should be conducted to find out suitable management for this complication.

## 1. Introduction

Rectus sheath hematoma (RSH) is a rare cause of abdominal pain in hospitalized patients. This condition commonly occurs after either a rupture of abdominopelvic arteries and their branches, especially during anticoagulation therapy or the tear of the rectus abdominis muscle [1].

Studies have warned against the development of RSH following anticoagulant treatment. It accounts for about 1.5–2% of the cases of unexplained abdominal pain in hospitalized patients [2]. The most common provoking factors are coughing, straining, exercising, hypertension, obesity, previous abdominal surgery, subcutaneous injection, prophylactic or therapeutic anticoagulation, and trauma. Trauma can be internal or external, including a strain of physical exertion, seizures, or even vomiting [3].

Clinical presentations of RSH include nausea, vomiting, fever, abdominal pain [4], and abdominal tenderness along with ecchymosis (Cullen's or Turner's sign) on physical examination [5].

Although a visible hematoma is a helpful sign in 50% of RSH cases [5], it can be easily screened by abdominal ultrasound (US) [2]; however, its diagnostic sensitivity for RSH is about 80–85%, and the results should be confirmed by abdominal and pelvic computed tomography (CT), which is 100% sensitive most of the time [6, 7].

There are concerns over RSH in hospitalized patients with COVID-19 [8–13]. On the one hand, these patients receive anticoagulants for thromboprophylaxis; on the other hand, any bleeding prompts clinicians to discontinue anticoagulants and control vital signs [14]. Therefore, surgeons can play an important role if bleeding progresses and vital signs are not controlled. Some predictors such as the rate of hemoglobin reduction, need for transfusion of packed red blood cells, bleeding confirmed by computed tomographic angiography, and hematoma size have been used to assess the need for angioembolization in recent years [15]. However, this complication and its clinical prognosis in COVID-19 patients are still unclear. This clinical retrospective multicentre study was conducted to shed light on ambiguities in this regard.

## 2. Material and Method

Hospital records were reviewed from June to August 2021 to extract demographic characteristics (age, gender, and BMI), past medical history, symptoms, laboratory tests, and imaging modalities supporting the diagnosis as well as treatment, duration of hospitalization, and RSH outcome.

A diagnosis of COVID-19 was made according to the chest computed tomography (CT) scan and/or real-time PCR upon admission. The severity of pulmonary involvement was assessed by a professional radiologist upon admission. RSH was diagnosed with sonography and confirmed by abdominopelvic CT in most cases after surgical consultation during the hospital stay.

Blood samples for coagulation state, inflammatory markers, and other routine tests were collected on hospital admission and repeated every day. During hospitalization, the dosing and method of anticoagulant administration were adjusted by clinicians at least twice a day based on inflammatory markers such as LDH, coagulation markers such as D-dimer, and vital signs (Table 1).

We present major RSH in nine confirmed COVID-19 patients on prophylactic or therapeutic anticoagulation treatment. The median and interquartile range were used to report the distribution of the results. Furthermore, written informed consent was obtained from the patients or their companions. This research was confirmed by the Research Ethics Committee of the Tehran University of Medical Sciences (approval id. IR.TUMS.VCR.REC.1399.462).

## 3. Results

About 24104 suspicious COVID-19 patients presented to respiratory triages, of whom 3820 were admitted. Nine cases were complicated with RSH.

The median age was 65 (22.5) years, most of them were females (F/M ratio: 8), the median length of hospital stay was 20 (13.5) days, and the length of anticoagulant treatment was 20 (13.5) days overall. The median BMI was 29.38 (7.74) kg/cm^2^.

The median levels of LDH and D-dimer on admission were 974.5 (517.75 to 1595) and 827.5 (567.5 to 1612.5), retrospectively.

Eight out of nine patients had abdominal pain and tenderness before hematoma, and eight had a cough as a minor trauma. One patient with a past medical history of myasthenia gravis had a right femoral catheter and received plasmapheresis four times before RSH. Since RSH in this patient could be due to the femoral catheter, considering the catheter as a confounding factor for RSH, her outcome was not included in the analysis.

It should be mentioned that all of the patients received antiplatelet drugs and steroids besides anticoagulation. All of them were on a daily dose of dexamethasone (8 mg) and acetylsalicylic acid (80 mg), and three of them with a past medical history of ischemic heart disease (IHD) received clopidogrel 75 mg. They continued to take their prescribed medications until a diagnosis of RSH was made.

All of the nine patients received subcutaneous injections of anticoagulant treatment at prophylactic or therapeutic doses as a routine ward practice. Six patients (66.6%) received therapeutic and others received prophylactic anticoagulation. Regarding the severity of COVID-19 at baseline, three (33.3%) hemodynamically unstable patients were already admitted to the intensive care unit (ICU) when RSH developed. Three others gradually became worse following bleeding and were transferred to the ICU. Another patient who was firstly admitted to the ward eventually died following an abrupt decrease in hemoglobin before ICU transfer.  Table 2 presents the details of all patients during hospitalization and their outcome.

Coagulation problems in most patients were corrected (achieving blood hemoglobin over 9 g/dL) with packed red blood cells (PRBCs). Three cases needed to receive fresh frozen plasma (FFP) as they had ongoing bleeding. More than half of the patients (66.7%) were admitted to the ICU. A 65-year-old female with moderate to severe confirmed COVID-19 developed a massive blushing hematoma following anticoagulant therapy, which was suspicious for extravasation on sonography. After receiving anticoagulation reversal procedures and fluid resuscitation, she was admitted to the catheterization laboratory (cath lab) for selective angioembolization in a tachycardia state before hypovolemic shock occurred. Selective angiography of the internal iliac and external iliac artery branches revealed no extravasation, and she did not require any special interventions on angiography.

Although appropriate surgical approaches were adopted including fluid resuscitation and correction of coagulopathy, five out of nine cases died. Hematoma resolved with conservative management in the youngest patients with moderate to severe COVID-19.

## 4. Discussion

Although RSH is an uncommon and self-limited cause of abdominal pain, it has become a new life-threatening concern among patients with COVID-19. While the mortality rate of RSH patients receiving anticoagulant treatment has been estimated at 4–25% in other patients [16], more than half of the cases in this study died despite receiving appropriate care in well-equipped referral hospitals (mortality rate was 55.5%). This rare occurrence is particularly associated with anticoagulant therapy [2, 17].

Considering the high prevalence of hypercoagulopathy state and the high risk of venous thromboembolism (VTE), anticoagulant therapy plays an important role, especially among hospitalized patients that are immobile due to severe pulmonary involvement. Reduced mobility, respiratory failure, obesity, and advanced age (>70) are predisposing factors for VTE as demonstrated in previous studies [18]. Besides, the prevalence of VTE has been reported to be higher in bedridden patients due to COVID-19 pneumonia, which can be predicted using D-dimer levels, especially in severe COVID-19 pneumonia [19]. In addition, studies have shown a higher incidence of pulmonary thromboembolism (PTE) in COVID-19 patients [20].

The role of laboratory markers such as D-dimer and LDH predicting the severe course of COVID-19 has been proven in recent studies [21], especially in thrombotic events [22]. For instance, an increased level of D-dimer can be a prognostic factor for bleeding or thrombotic complications, critical illness, and death. The elevated LDH is identified as a risk factor for thrombosis in patients with COVID-19 as well [22, 23]. Besides, LDH has long been an early inflammatory marker of hemolysis to predict thrombosis in patients with ventricular assist devices in articles [24, 25].

Since the COVID-19 can intervene in intravascular systems, the majority of all deaths in patients with COVID-19 are attributed to VTE and disseminated intravascular coagulation (DIC) which are consequences of the systemic inflammatory response [26, 27]. PTE is also associated with marked changes in some laboratory markers such as D-dimer and LDH according to recent radiologic surveys [28, 29], that is why proper anticoagulant treatment to reduce PTE complications in COVID-19 patients is so crucial.

The optimal dose of thromboprophylaxis has yet to be determined; therefore, in this study, any decrease in blood oxygen despite routine treatment made the clinicians switch from a prophylactic dose to a therapeutic dose. Although none of the patients in this study had confirmed VTE/PE on CT imaging, clinicians are interested in anticoagulant therapy considering recent studies endorsing a reduction in the use of respiratory or cardiovascular organ support following the administration of therapeutic doses of anticoagulants [30]. Besides, the rising trend of D-dimer and LDH at the same time was also suggestive of VTE or PTE in these acutely ill patients.

Although thromboprophylaxis, if not contraindicated, has been proved to be beneficial in hospitalized patients with COVID-19 [31, 32], there are reports of RSH [33] in these patients [8, 9].

Considering a recent Canadian report indicating a chance of about 5.6% for major bleeding in these patients [34], five patients in this study received therapeutic anticoagulation treatment according to the American College of Chest Physicians guidelines [35], and the others received prophylactic doses instead as a routine ward practice until a diagnosis of rectus sheath hematoma was confirmed [36]. Even though some studies have warned against LMWH (enoxaparin) [33], most of the cases received this drug due to its availability and reasonable price. Comorbidities such as hypertension (HTN) and diabetes mellitus (DM) as well as high D-dimer levels on admission have been considered as factors associated with a poor prognosis in COVID-19 patients [37] (Table 2).

Despite all efforts to overcome blood loss, restore stable vital signs, and stabilize RSH without any reduction in blood hemoglobin (over 9), the mortality rate was more than expected among these COVID-19 patients (55.5%). It seems that due to the deterioration of the underlying viral disease, their condition became worse unexpectedly.

According to similar studies, most of the cases in this study had multiple risk factors for RSH, which increased the risk of RSH during hospitalization [38]. All cases, except one, were female, and female gender is a major predisposing factor for RSH. Except for one with femoral catheterization, half of the other COVID-19 patients (50%) were admitted with a gastrointestinal presentation (nausea, vomiting, diarrhea, abdominal pain, etc.). All patients received subcutaneous injections of anticoagulants in the abdomen, and most of them (87.5%) had at least either abdominal pain or cough right before RSH. By paying timely attention to these clues, clinicians can manage this fatal complication more properly.

## 5. Limitation

Since central venous catheterization is associated with a risk of bleeding in medical patients [39], the patient with myasthenia gravis who developed RSH after receiving plasmapheresis through the femoral catheter was not included in the analysis.

Two ultrasound reports containing information on the size and location of RSH were not available; therefore, they were only described qualitatively.

## 6. Conclusion

Although RSH is usually self-limited and has a good prognosis, it can be a life-threatening complication in COVID-19 patients receiving anticoagulant treatment. Hospitalized COVID-19 patients with RSH risk factors should be identified on admission, and healthcare providers should bear in mind the utmost significance of an abrupt decrease in the hemoglobin level together with an increase in inflammatory markers such as LDH in these patients during daily visits. Further studies in larger sample sizes are needed to find better management options for COVID-19 patients with RSH.

## Figures and Tables

**Table 1:** COVID-19 severity and vital sign summary in admission time.

Patient number	COVID-19 severity	O_2_ SAT (%)	SBP	DBP	Max Hb	Min Hb
1	Severe	87	140	75	11.8	8.9
2	Severe	75	140	85	10.5	8.6
3	Severe	75	116	65	11	8
4	Moderate to severe	67	110	70	12	7
5	Moderate to severe	94	140	80	14.4	6.8
6	Moderate to severe	85	120	80	13	9
7	Moderate to severe	89	170	85	12.9	5.7
8	Moderate	70	110	70	13.5	7.5
9	Mild to moderate	86	110	70	11	8.5

SBP: systolic blood pressure (mmHg); DBP: diastolic blood pressure (mmHg); Hb: hemoglobin; O_2_ SAT (%): oxygen saturation (without oxygen support); Max: maximum; Min: minimum (bleeding time).

**Table 2:** Study case summary.

Patient	BMI (kg/m^2^)	Past medical history	Laboratory findings			Anticoagulant	Complication					Admitted to	Outcome	
			CRP	D-dimer	LDH	Regimen	Total received days	Total received dosage	Location of RSH	Size of RSH	Management		Total hospitalization stay	Final status
A 78-year-old female	27.34	HTN	99	1410	2300	LMWH (therapeutic dose)	14	60 daily	Unilateral massive RSH	Unclear	PRBC	ICU	20	Expired
A 61-year-old female	33.30	None	124	NA	1600	LMWH (therapeutic dose)	15	60 BD	Bilateral expanded to hypogastric zone and free fluid in the pelvic	64∗89 mm	PRBC	ICU	37	Expired
										59∗29 mm				
										76∗22 mm				

A 78-year-old female	37.00	IHD, HTN, and arrhythmia	120	685	482	UFH (prophylactic dose)	5	5000 TDS	Unilateral massive RSH	Unclear	PRBC	ICU	6	Expired
A 65-year-old female	28.30	DM	71	2735	1183	LMWH (therapeutic dose)	10	60 BD	Infra oblique hematoma, expansion to pelvic with probable extravasation, and free fluid in the abdomen	Maximum size of 170∗155∗90 mm (150 cc)	PRBC, FFP, fibrinogen, and factor VІІ infusion	Ward, then ICU and cath lab	13	Expired
An 83-year-old female	20.60	IHD and asthma	12	1680	1580	LMWH (therapeutic dose)	7	60 BD	Massive bilateral RSH	maximum size of 130∗65 mm	PRBC	Ward	7	Expired
A 43-year-old female	29.74	DM	85	650	625	LMWH (therapeutic dose)	9	60 BD	Left unilateral RSH	326∗92∗42 mm	Conservative	Ward, then ICU	26	Discharged
A 67-year-old female	25.39	HTN and IHD	142	540	465	LMWH (therapeutic dose)	14	60 BD	Retroperitoneal and hypogastric RSH with expansion to pelvic	110∗108∗128 mm in size (790 cc)	PRBC and FFP infusion	Ward, then ICU	20	Discharged
A 50-year-old female	29.38	HTN, DM, and MG	110	970	766	LMWH (therapeutic dose)	10	60 BD	Bilateral RSH with expansion to latissimus dorsi	Maximum size of 134∗67 (330 cc)	PRBC and FFP infusion	Ward	21	Discharged
A 62-year-old male	19.60	DM and KT	68	450	NA	UFH (prophylactic dose)	5	5000 TDS	Retro rectus hematoma in latissimus dorsi and free fluid in the abdomen	Not lobulated, volume/size was not detectable	PRBC	Ward	17	Discharged

BMI: body mass index; HTN: hypertension; DM: diabetes mellitus; IHD: ischemic heart disease; MG: myasthenia gravis; KT: kidney transplant; CRP: C-reactive protein; LDH: lactic acid dehydrogenase; ICU: intensive care unit; UFH: unfractionated heparin; LMWH: low-molecular weight heparin (enoxaparin); BD: twice a day; TDS: three times a day; PRBC: packed red blood cell.

## Data Availability

The data used to support the findings of this study are available on request.
